# What’s Next in Design for Global Health? How Design and Global Health Must Adapt for a Preferable Future

**DOI:** 10.9745/GHSP-D-21-00280

**Published:** 2021-11-29

**Authors:** Ayush Chauhan, Krista Donaldson, Ana Santos, Michael Ngigi

**Affiliations:** a Quicksand, New Delhi, India.; b Equalize Health, San Francisco, CA, USA.; c ThinkPlace Kenya, Nairobi, Kenya.; d PATH, Nairobi, Kenya.

## Abstract

Integrating the practice of design with global health offers a way to ensure that all voices—from patients to policy makers—are all heard in conceiving and developing solutions that address the current misalignments and support efforts to make quality health care more affordable, accessible, and humanized for all.

## Key Messages

- Design for global health is an emerging field of diverse histories and areas of misalignment in creating equity. Using a health futures framework, it is possible to better align incentives and strategies to improve the effectiveness of global health efforts. To do so, will require shifts in mindset and practice in both global health and design.
- The COVID-19 pandemic and challenges to scaling sustainable solutions have highlighted the need to evolve both practices, for example, recognizing the interdependence between planetary and human health and refocusing our efforts on the health seeker instead of merely on the systems and technologies for health provision.
- For a preferable future in which there is greater coordination and improved health outcomes even in emergencies, the global health sector must evolve to an inherent focus on the health of individuals over health care. Design practice must evolve itself toward new paradigms of life-centered design and speculative design.

## INTRODUCTION

Our global society faces unprecedented challenges and demands for appropriate, rethought solutions to how our lives are organized. The pandemic future predicted by experts^1^^–^^3^ arrived in late 2019 with the coronavirus disease (COVID-19)—a period where society witnessed collaborative global health in action. While there were many successes in the global response, there were also significant areas for improvement that could have saved lives and significantly increased well-being.

In this commentary, we collectively envision the future of design in global health and the interactions between the disciplines. We do this to inspire and strengthen the practice of design for global health and to provoke the continuous development of this thriving and emerging field. To address the needs and opportunities of our future, our sector must also recognize the influences of our past and the nascent stage of design for global health today. Our commentary is meant as acknowledgment and encouragement to all those working to create health equity.

## A BRIEF HISTORY OF DESIGN FOR GLOBAL HEALTH

Design for global health represents a convergence of relatively new fields, many still defining themselves while continuously evolving in response to new challenges.

While public health efforts date back to quarantine practices to prevent the spread of the bubonic plague in European states in the 14th century,^4^ definitions of global health relative to public health and international health started in earnest in the late 2000s.^5^^–^^7^ Despite global health’s recent emergence as a unique discipline^8^ “the central motivations, organizing principles, and modes of operation that characterize it” hark back to its colonial roots in military medicine and international health.^8^ The focus then was on infectious diseases of white settlers and laborers rather than health services that served the broader population.^8^ Furthermore, strategies were “imposed from above with little concern for the ideas or the cooperation of local residents.”^8^

The best-known example is U.S. Army physician William Gorgas’s efforts to eradicate the *Aedes aegyptei* mosquito that transmits yellow fever, first in Cuba (1898) and then Panama (1904) to protect workers excavating the canal. Gorgas and others coordinated disease-control strategies while establishing new university programs in tropical medicine and hygiene in the United States and Europe that set the path for future and current global health priorities.^8^

A more equitable view of health was first established at the Pan-African conferences in Cape Town (1932) and Johannesburg (1935) and later with the creation of the World Health Organization (WHO) (1946). A broad definition of health was reaffirmed with the 1974 Alma-Ata Declaration by delegates from 134 countries.^9^ Yet, global health programs continued to operate in top-down verticals with limited engagement with local populations; their health was largely the domain of medical missionaries from Europe and North America.^8^ Despite significant progress within the field, the lack of equitable engagement cannot be dismissed as an artifact of previous centuries: high HIV prevalence and low condom usage in sub-Saharan Africa in the early 2000s were blamed on people rather than the pushed global health “solution” (condoms).^10^^–^^12^

Design for global health—realized largely in the implementation of design methods in the development of global health products and services—started in earnest in the mid-2000s arriving via 2 streams of work. The first stream was “design for development,” later called “design for social impact,” which can be traced back to the appropriate technology movement (late 1960s through 1990s), and the writings of globally-minded industrial designers.^13^^–^^15^ The Ahmedabad Declaration on Industrial Design for Development in 1979 was notable in that it represented the^16^ “first plan of action agreed upon by designers **from less-developed countries**” (emphasis added).

The Development by Design conference in 2002 in Bangalore, India, marked the emergence of “design for good” programs at universities and small nongovernmental organizations. The second stream came as design thinking gained traction in academia and business: in the 1990s, hospital systems and medical device companies started employing designers, who are generalists by nature, to not just improve products but strengthen patient awareness and engagement.^17^ Uptake and integration of design into global health practices, however, has been relatively slow compared to other fields and industries. We hypothesize that this may be in part due to the range of decision makers, the complexity of stakeholder relationships and business models, and the dominant therapies orientation that prioritizes medical and academic specialists’ perceptions rather than consumer voices. The transition from design as a set of tools (to add value to a project) to an ethos fully integrated throughout strategy and process (that drives change in large complex systems) is just beginning and far from complete.

## DESIGN FOR GLOBAL HEALTH NOW

Design’s core principles around setting strategy based on empathy and the user’s voice, investing deeply in understanding context, “failing fast” and iterating, and end-to-end problem solving are highly applicable to addressing challenges of global health.^18^^,^^19^ Over the last decade, we, as practitioners of design for global health, have been encouraged by the increasing changes in the field’s practice to promote empathy, equity, and inclusion. A simple example is the transformation in language norms from “beneficiaries” to “patients” or “users” to even “customers.” This evolution reflects broader shifts in perspective that increasingly recognize choice and one’s role in their own health care—as well as the necessity of implementation strategies that prioritize sustained impact.

Although the health of many people has improved in the last decade, pre-pandemic data indicated that none of the United Nations Sustainable Development Goals in health will be met by 2030.^20^ Yet, many of the preventable diseases that continue to wreak havoc among low-income populations are well understood with existing solutions. For example, continuous positive airway pressure therapy was first used on neonates in the early 1970s,^21^ but respiratory distress syndrome remains a leading cause of infant mortality in low- and middle-income regions today.^22^

Although the health of many people has improved in the last decade, data indicate that none of the United Nations Sustainable Development Goals in health will be met by 2030.

As a field, the range of necessary changes to drive broad health equity has not been implemented. As practitioners, we observe present-day misalignments in design for global health: under-investment in understanding context and constraints before strategy and concept development, deterrents to discuss “failures,” insufficient consideration of sustainable implementation and scaling, a “good enough” mentality resulting in low innovation quality, and short-term funding horizons that discourage iteration critical to creating impact. These incongruities are not new.^23^ In considering the future of global health and how design can best be integrated, our sector must acknowledge and address the misalignments as well as the challenges of the present: managing the increase of noncommunicable diseases, preparing systems to respond to emerging diseases, planning for growing urbanization and planetary change, and supporting efforts to make quality health care more affordable, accessible, and humanized for all. However, we, true to nature, are optimistic: design provides us with the means to solve such complex challenges.

The practice of design for global health offers a means to ensure all voices—patients, families, health care providers, maintenance technicians, cleaners, distributors, policy makers, responsible government officials, and others—are all heard in conceiving and developing solutions that range from devices and physical work environments to innovative strategies and improved services. Design offers invaluable tools to deeply understand problems and related stakeholders, adapt and develop solutions, as well as anticipate and prepare for the future.

Design offers invaluable tools to deeply understand problems and related stakeholders, adapt and develop solutions, as well as anticipate and prepare for the future.

## THE FUTURE ROLE OF DESIGN IN GLOBAL HEALTH

The COVID-19 pandemic, as disruptive as it has been, has offered an opportunity for the global health community to reinvent itself, asking once again certain fundamental questions around equity, inclusiveness, and diversity. Design, which has been largely on the edges of global health, offers utility in this transformation. As a community, we must ask: How can design best contribute to the next wave of health revolution while also transforming itself in ways that it is equipped to address the new and unprecedented challenges in the health sector?

**FIGURE 1:**
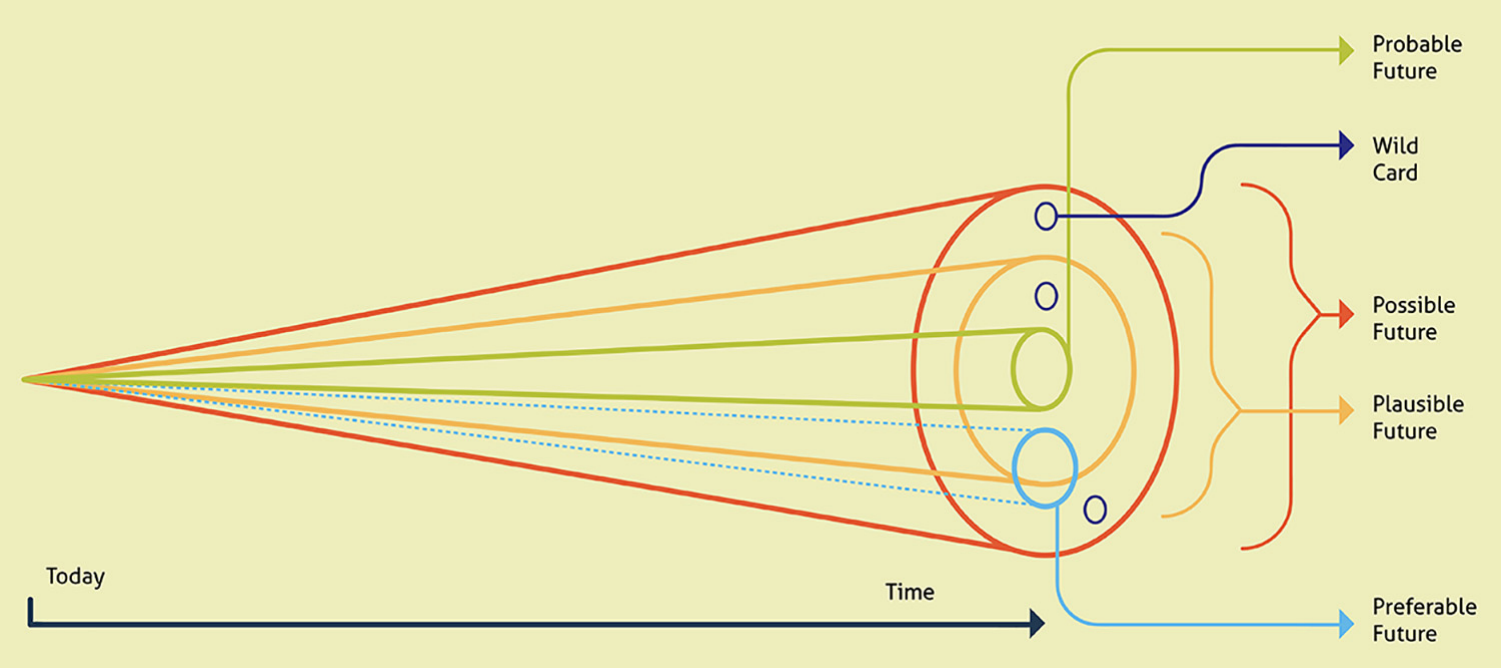
The Futures Cone: Envisioning Various Futures for Design in Global Health^24^

To answer this question, we refer to a framework proposed by Hancock and Bezold^24^ to explore health futures (Figure 1). They note^24^:

*at its best, health futures work does more than what might happen; it enables individuals and organizations to find or enhance the leadership necessary to move in desired directions.*

Consequently, they infer that futurism is much less an attempt to accurately forecast the future as it is a call to organizations and communities to recognize that the “future is plastic” and therefore can be shaped if institutions and individuals have an imagination for it.

Using Henchey’s typology of futures,^25^ there are 4 ways of thinking about the future:

1. **Possible future** (what may be) encompasses everything we can possibly imagine, no matter how unlikely.
2. **Preferable future** (what should be) is what we want to have happen and falls in the realm of organizational or societal visions that “move reality beyond the present toward the best that can be.”^24^
3. **Plausible future** (what could be) is a range of alternative futures based on what we know today, combining differing trends and scenarios.
4. **Probable future** (what will likely be) is what will likely happen and is based on examination of our current situation and an appraisal of likely trends and future developments.

As practitioners and researchers, we base our assertions about the future of health by examining these possibilities through a design lens. Notably, our attempt is not to be predictive but to claim our agency as a community in determining a future that is informed by the natural proclivities, developments, and imaginations of design practice.

Our proposition for the future role of design in global health is 3-fold. These futures play out over different time horizons and indeed align well with the different typologies in Figure 1.

### 1. A Preferable Future That Shifts the Focus From Health Care Back to the Health of Individuals Through User-Led Design

Design advocates for the patient’s well-being to be at the center of health care provision, with health care providers—also users—in support. We have seen health sectors challenged by misaligned incentives and complex systems that have resulted in the individual pushed outside of the core mission of “health for health’s sake.” For example, privatization of health care in many markets has created an incentive structure that financially benefits from a continuously unwell population rather than a healthier trendline. Within months of the start of the COVID pandemic, mid-to-high tier private hospitals in India were reporting as much as a 90% decrease in revenue because of the “sharp drops” in outpatient visits, elective surgeries, and international patients.^26^ India is not the only country.^27^^,^^28^ Although global health funding patterns are starting to shift, the development of solutions has traditionally been driven by academic institutes that are removed from the contextual and market realities of target users. The results, as might be predicted, are underwhelming at best: an analysis of funding for maternal and newborn health innovation found that the largest slice, more than 40% (∼US$90.7 million), went to universities largely in high-income countries and none of those innovations scaled to a level of sustainable impact.^29^

Prioritizing the short-term as well as long-term needs of health care seekers can shift the future arc of health systems by participating in the achievement of the desired outcomes or preferred future. Giving greater ownership to users too will shift incentives from financial good to social good where solutions are developed by or in collaboration with the individuals and communities the solutions seek to serve.

Prioritizing the short- and long-term needs of health care seekers can shift the future arc of health systems by participating in the achievement of the desired outcomes or preferred future.

**FIGURE 2:**
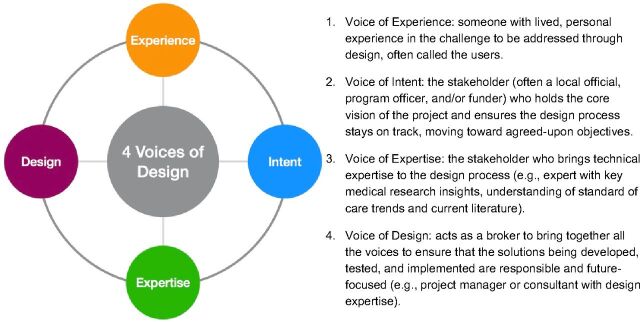
The Four Voices of Design^a^ ^a^ Adapted from ThinkPlace.^30^

User-led health care can be found in initiatives such as the creation of youth advisory committees that select community health workers to serve them, the purposeful integration of persons with disabilities in the design of targeted health programming, and the financial support of multidisciplinary community labs that identify, prioritize, and address their own problems. Through guided co-creation,† design can support global health strategists and decision makers to define and understand the key problems, develop the right goals, and implement preferable systems that promote equity and health improvement. This co-creation process consists of several steps that require different levels of involvement from each of the different voices of design (Figure 2) that represent the critical stakeholders required to develop solutions that are human-centered and sustainable.^30^ A strong predictor of failure in design processes is where 1 or more of these voices is weak or missing.

**Figure:**
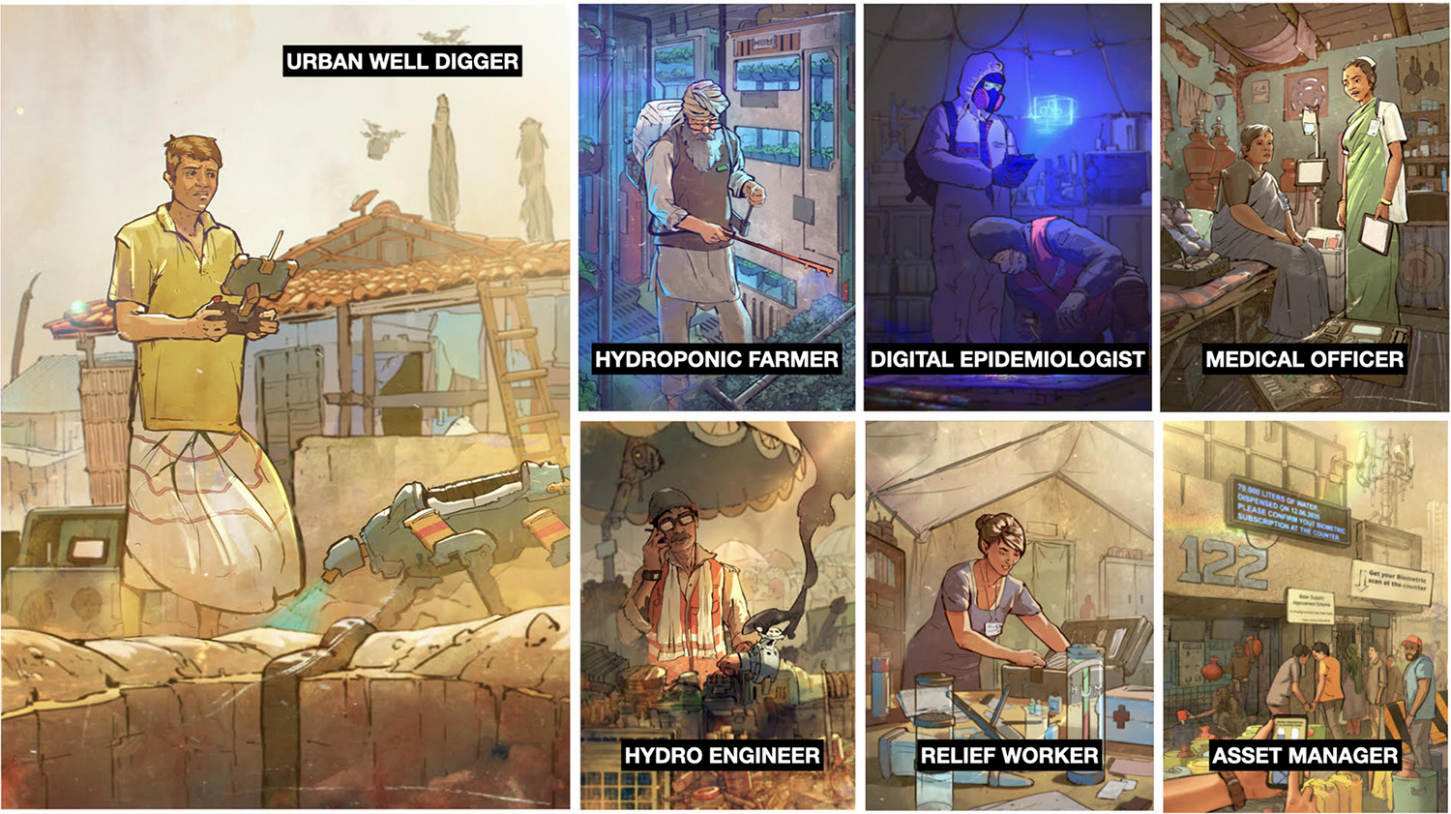
HUM.2035 personas of future humanitarian workers addressing a water crisis in South Asia. © 2019 Quicksand

**Figure:**
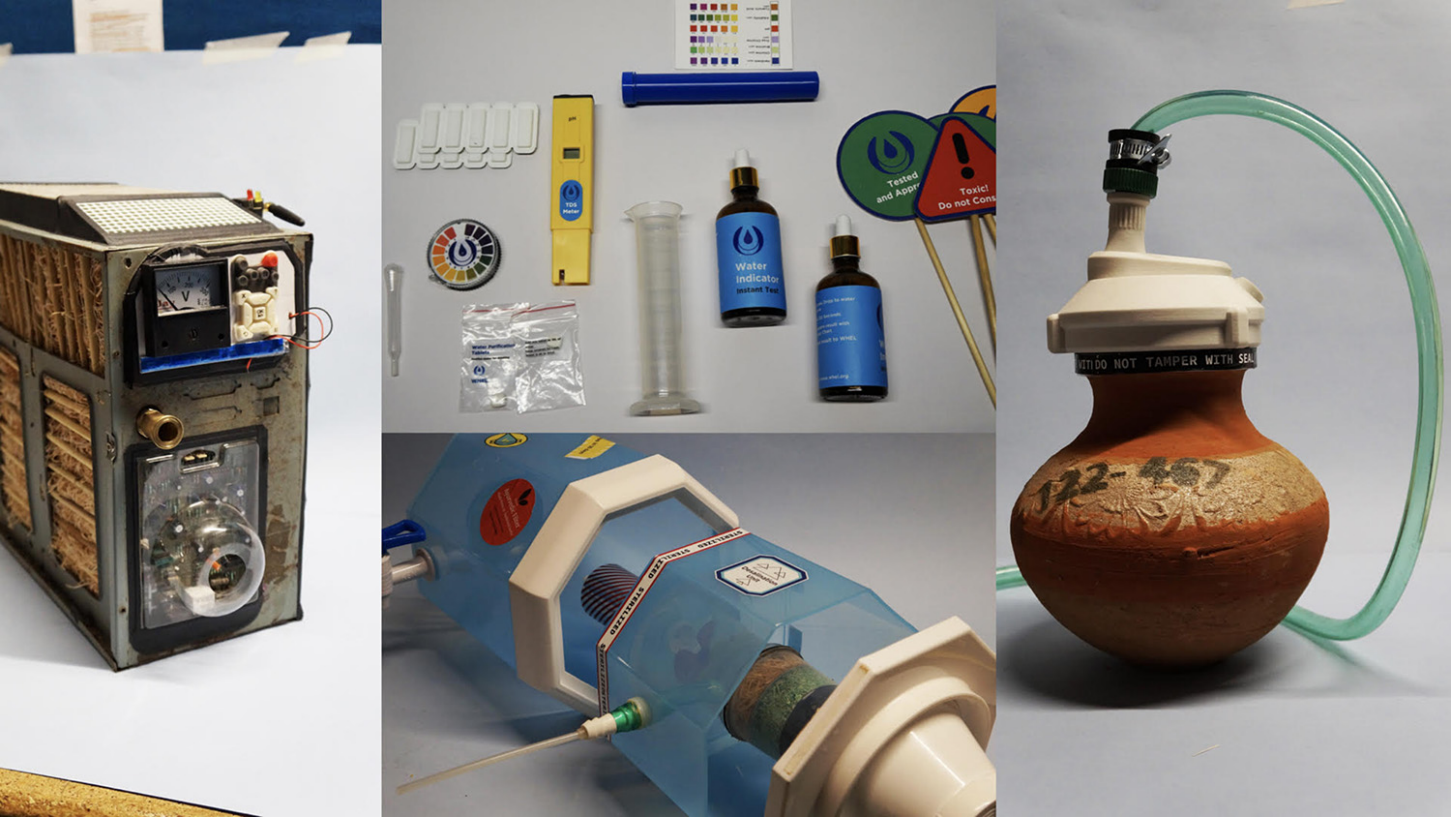
Prototypes of objects and artifacts from the future that became a part of the HUM.2035 exhibition. © 2019 Quicksand

**Figure:**
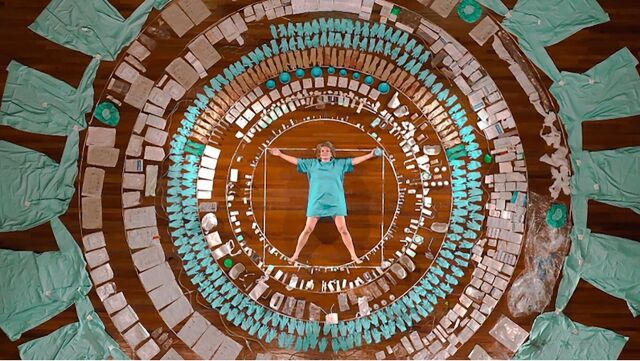
Dutch artist Maria Koijck collected the medical waste from her breast cancer operations. Sixty percent of the materials were disposable. “Is there another way?” she asked.^48^ © 2020 Marja Koijck

**Figure:**
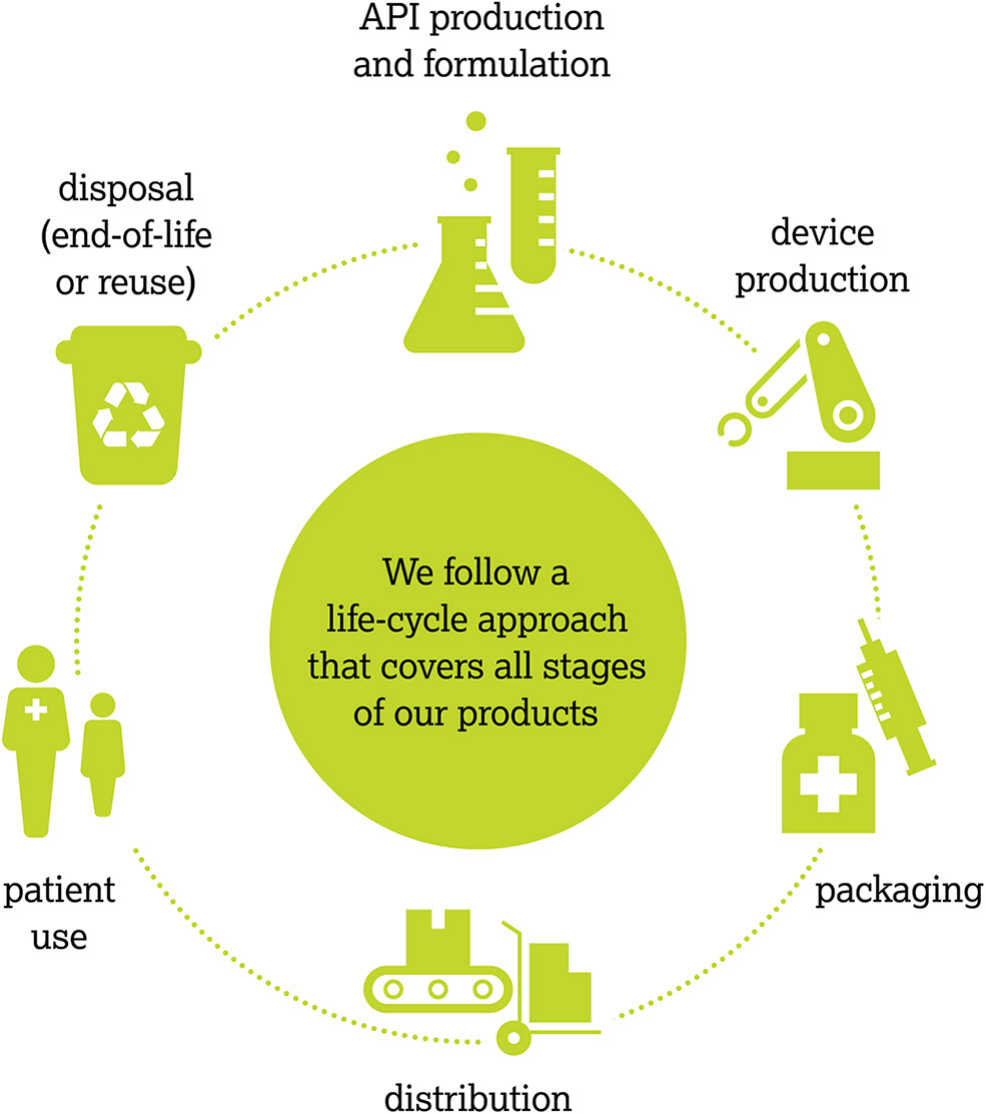
AstraZeneca’s staged life cycle approach in pharmaceutical development (API is active pharmaceutical ingredient). © 2020 AstraZeneca

For a preferred future, the design process engages the 4 voices of design in the following ways:

- **Defining the challenge** to be addressed, the range of stakeholders and users, and who they believe are the ultimate customers, as part of the **intent phase**. This exercise (e.g., guided conversation and customer value chain analysis^31^) emphasizes articulating the desired future state to the challenge.
- **Gaining a deep understanding** of the challenge, context, and people involved. This **exploration phase** must include learning of the experiences of the people who are closest to this challenge to appreciate the complexities of the challenge (“voice of experience”). The “voice of design” leads the group in developing user archetypes to move into the next phase with targeted briefs that address specific personas.
- Rapidly **generating prototypes and testing new ideas** that speak to the defined challenges. This is an iterative process based on stakeholder input that works on the idea that one should “fail fast and learn fast” to get to a desirable, viable, and feasible solution to the challenge being addressed. Here the voice of design works with the users (“voice of experience”) and subject matter experts (“voice of expertise”) to identify and test potential solutions. Throughout this process, all potential solutions are evaluated, and ultimately the most promising solution is selected for implementation, as part of the **innovation phase**.
- **Iterating purposefully** to ensure the selected solution performs as intended in real environments while meeting other requirements for economic viability, scaling, sustainability, and impact. This **implementation phase** does not end once a first solution launches but should extend over time as the solution’s effectiveness is evaluated.

We offer 2 examples of approaches that sought a preferred future with preventative health initiatives: 1 that did not work, and 1 that did.

Preventive health care efforts to date have been largely prescriptive and fear-based.^32^^,^^33^ These approaches have been controversial, often with poor outcomes,^34^^–^^37^ owing to lack of “voice of experience” and dearth of prototyping, learning, and timely iteration. In Kenya, when HIV/AIDS was declared a national threat in 1984, fear-based messaging was the initial response of nongovernmental organizations and the government. Most of the preventive health communication at the time featured imagery of wasted and dying people to influence behavior change. It was largely unsuccessful as the virus spread at an unprecedented rate. Other aspects that influence people’s behaviors, such as environment and sociodemographic factors, were inadequately factored into campaigns resulting in avoidance responses among those for whom the health threat was self-relevant.^38^

Some preventive health care approaches have often had poor outcomes because of the lack of “voice of experience,” prototyping, learning, and timely iteration.

Our experience from “voice of experience” and user research across a range of geographies suggests that preventative health care initiatives are more successful when they offer opportunities that fit well with people’s lifestyles, constraints, and schedules; they promote social interactions; and they provide positive reinforcement. Studies have shown successful outcomes with community programs that increase participation in one’s own wellness management. For example, a U.S. study showed that no-cost gym visits are associated with lower weight and blood pressure among African American and Latinx participants who have hypertension.^39^ In Dakar, Senegal, the installation of workout equipment on beaches led to the rise of a social fitness movement that has sustained momentum for more than 20 years. Localized programs grew from that movement, such as group exercise classes where participants also socialize, encourage, and connect.^40^ In both locations, the delivery vehicle for preventative health (accessible gyms) met users’ needs and fit within their lifestyles without significant undesirable behavior change.

### 2. A Plausible Future That Leverages Design’s Collaborative and Continuous Learning Principles to Encompass Ecosystems

The WHO’s constitution^41^ recognizes with its first line:

*Health is a state of complete physical, mental, social, and ecological well-being and not merely the absence of disease or infirmity*

that we share more than our genome: we share the air we breathe, and ultimately vulnerability to our environmental context. Planetary health and holistic health, fields introduced decades ago, extend the concept of medicine to safeguard the health of persons, places, and our planet by acknowledging interdependencies between the planetary life-support systems and the health of individuals.^42^ While modern global health seeks to strengthen health broadly and create equity at the population level, these life-centered concepts call for a wider look at how humans affect and are affected by our shared environment, infrastructure, and ecology. This broader conceptualization suggests that indirect solutions exist and are potentially more effective to address today’s as well as tomorrow’s burden of diseases.

Designing for planetary health calls for global health practitioners to look beyond social determinants to include environmental determinants. As we consider the future, 10 billion people will be breathing, “eating, moving, plugging in, building, buying, using, wasting, and all the rest in 2050.” ^43^

Designing for planetary health calls for global health practitioners to look beyond social determinants to include environmental determinants.

We as a global society will be challenged by new diseases affecting people, animals, and plants, as well as recurring diseases in different forms and locations. Global health needs to provide leadership in a greater range of domains, including food systems, community preparedness, politics, and urban planning.

We suggest that a plausible future focuses on improving the health of human beings and the planet in union with a focus on resilient and large-scale solutions. A renewed discourse calls for thinking beyond human-centered design practices to the inclusion of ecological and environmental requirements in design methodologies.^44^ **Life-centered design (LCD)** borrows principles and tools from design for sustainability, circular design, and biomimicry to offer an approach to product, service, and systems design that encompasses people, profit, and the planet at its core.^45^^–^^48^

Given the early stage of LCD as a practice, as well as the complex nature of systems thinking, different disciplines have developed and adopted a range of principles and tools. We offer the following resources, best layered, to integrate LCD when developing strategies and solutions.

- **Product lifecycle analysis**^49^ applied throughout the solution development process has become a key requirement for production systems that reuse, reduce, and optimize natural resources, materials, energy, and emissions. Many industry actors, including those in health care, such as AstraZeneca, also leverage design tools (e.g., design for modularity, design for disassembly, and design for repairability)^50^ and/or metrics to support life cycle considerations and improvements (e.g., process mass intensity, energy productivity, and percentage of sustainable sourcing for packaging materials).^51^^,^^52^
- **Product-service systems** are business models that “deliver value in use,”^53^ enabling organizations delivering products to extend their value by offering related services.^54^ Product-service systems approaches aim to enhance the value of their commercial products while reducing their environmental impact and harmful footprint. For example, a point-of-care testing device such as a glucometer might offer reminders for testing or automatically transmit unexpected results to a clinician, reducing waste and saving energy.^53^
- **Regenerative** **design** practices focus on restoring or revitalizing sources of energy and materials at the scale of institutions and the built environment by better embedding designs in the natural world. Butaro District Hospital in Rwanda was designed to mitigate and reduce the transmission of airborne disease by leveraging indoor and outdoor space, the surrounding geography, and natural cross-ventilation.^55^ Other examples include green roofs, thermal efficiency, and actively sustaining surrounding natural habitats.

Our list and examples are not comprehensive. When layering LCD processes, we realize that the degree of change, timeframe, and the number of stakeholders that are required to enact the needed transformative innovation is great, but they are not insurmountable. Change must be driven at the institutional level by global health’s leading “voices of intent” to best affect the fundamentals of society, including industry and sectoral norms and values, sociocultural practices, and economies. The cost to implement LCD practices must be viewed as a necessity to remain relevant beyond the short term;^56^ organizations must address current challenges while continuing to adapt to the plausible future of global health.

Change must be driven at the institutional level by global health’s leading “voices of intent” to best affect the fundamentals of society, including industry and sectoral norms and values, sociocultural practices, and economies.

### 3. A Possible Future That Rallies the Co-creative and Imaginative Powers of Individuals and Institutions Through Speculative Design

In the parlance of futurists—those that systematically explore predictions and possibilities about the future by studying current trends and realities—the COVID-19 pandemic represents a wildcard moment, a “possible” future that was imagined but ignored and as a result upended global systems. The need to truly operate with resilience and responsiveness at a global scale, and in a manner that is equitable, inclusive, and radically collaborative has never been greater. It is also evident that the trajectory of global health is impacted by factors that were either missing from conversations on health systems design or did not force a reckoning as directly and urgently.

For example, the tension between sovereignty and collective global action against a shared threat, such as epidemic outbreaks, has been an oft-debated point around governance in global health.^57^ Especially in low-income countries, the state's ability to protect and promote the right to health is eroded by trade and investment treaties that privilege investors over governments and provide avenues for international corporations to challenge democratically enacted public health policies.^58^ It may be argued that the disparities that COVID-19 has revealed and created, in terms of access to care and supplies such as oxygen and vaccines, could further exacerbate the perceptions of global health as neocolonialist and tip the debate in favor of stronger sovereignty over health care.

At the same time, technology is aiding more regional and local self-sufficiency. India’s National Health Stack envisions a digital infrastructure designed as a common public good accessible by central and state governments and public and private sectors.^59^ It is expected that the National Health Stack will become the cornerstone for India’s public health system, as well as offer a model for other emerging economies.

We are in a period of tectonic shifts that will have a deep and lasting impact on global health. Therefore, it is not enough to engage in conversations around “what is likely” but be wildly more imaginative in asking questions around “what if” to prepare for the social, cultural, technological, environmental, and political shifts that will eventually determine the future of human and planetary health. Incidentally, design is well-positioned to facilitate these conversations. A particular method of speculative design achieves this by creating scenarios and experiences from a set of imagined futures that stakeholders can engage with and debate its merits and flaws. The creativity inherent in design lets us transcend the known into the unknown, allowing us to be critical of what is currently happening and to reflect on the direction in which we are heading. Returning to Hancock and Bezold’s health futures work, they argue that doing speculative work forces a reckoning with the values of individual actors and the community—an aspect that, both the history and current state of global health reveals, has long been overlooked.

We are in a period of tectonic shifts that will have a deep and lasting impact on global health.

In the humanitarian sector, for example, speculative design has been used to help organizations consider the future of their work in a rapidly changing world. HUM.2035 is a project that developed forecasts for how humanitarian work would fundamentally change based on the shifting nature and location of crises caused by climate change, livelihoods disruption, geopolitics, digitalization, et al. The process used tools and techniques from the field of speculative design.

- **Investigating** the current humanitarian ecosystem, including forces influencing humanitarian work
- **Imagining** spaces, events, and scenarios in the future that are manifestations and extrapolations of these shifts
- **Bringing to life** future scenarios through immersive media, videos, interactive exhibits, illustrations, and physical artifacts that serve as a provocation for how the humanitarian ecosystem was changing
- **Engaging** specific institutional actors and leaders to consider these future shifts in their ongoing organizational transformation and change management agendas

HUM.2035 (HUM represents the word “we” in Hindi) tells the stories of humanitarian workers dealing with the aftermath of a devastating tropical storm Lata that has hit Goa in the year 2035. HUM.2035 started as a traveling exhibit on the future of humanitarian work, first displayed in London.^60^ Médecins Sans Frontières leveraged the exhibit to facilitate internal strategic conversations with more than 80 heads of missions and medical coordinators on the future of the organization. The power of this tool became evident as conversations shifted from talking about power (who is going to make decisions and where) to value creation (how the organization must rethink its services given the changing nature of crises).

**FIGURE 3:**
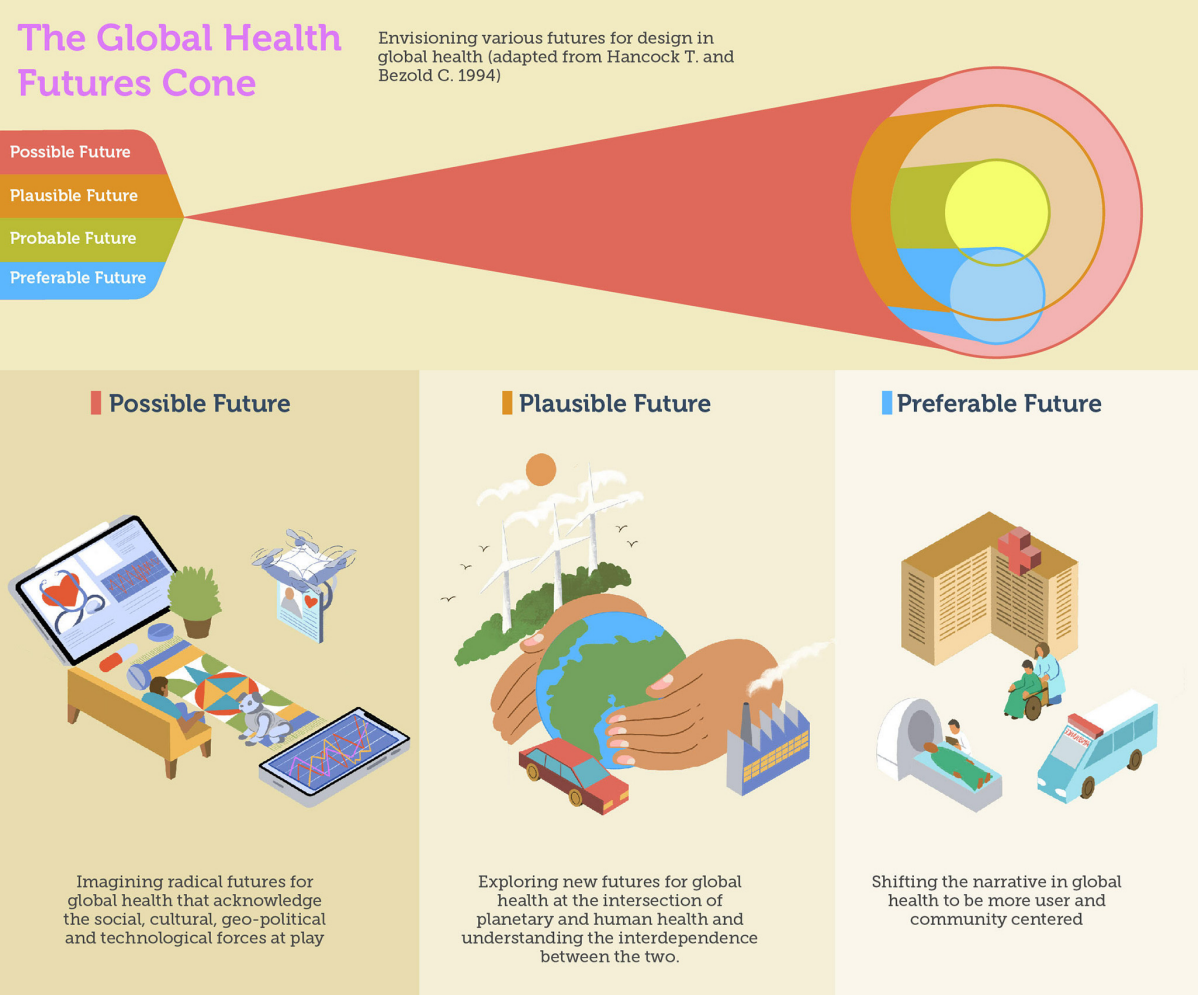
The Global Health Futures Cone to Consider Possible, Plausible, and Preferable Futures Based on the Work of Hancock and Bezold.^24^

In the context of global health, a speculative design process would involve looking at adjacencies to health that do not figure prominently in the discourse at the moment but may have a strong influence on how health care is sought and delivered in the coming decades. Trends such as the shifting center of geopolitics; emergence of big tech and concerns around data privacy; genomics and related ethics; and linkages between health, financial, and climatic vulnerabilities of communities will point to a range of possibilities around how health care is transacted in the future. Speculative design brings various actors together, helps conceptualize the future, and facilitates conversations on the values, intent, and directions that current systems and organizations are designed for and how they must be recalibrated (Figure 3).

A speculative design process entails looking at adjacencies to health that do not figure prominently in the current discourse but may strongly influence how health care is sought and delivered in the future.

## CONCLUSION

It is evident from our current vantage that the future of global health will require the simultaneous exploration of possible, plausible, and preferable futures—from the highly unlikely developments that can upend our notions of health care to those instances where the link between planetary and human health is clear for moments when giving privilege to the voice of the end consumer and communities is the prudent, ethical, and equitable thing to do.

Global health, as a sector, has the knowledge and experience of history and diverse societies to address health challenges throughout the world. Design enables us to better act on this knowledge, engaging both users and communities to develop lasting sustainable solutions that create equity and impact. Design can best contribute to reform the next wave of health revolution by addressing misalignments in existing global health practice and shifting focus from health care to health of broader ecologies. The practice of design too therefore must evolve to push beyond traditionally defined sectoral systems to the larger global ecosystems, complimenting user-centered design with LCD. Finally, the collaborative, ambitious, and speculative nature of design offers the means and tools to pre-empt changes at the level of the individual, communities, and society that will fundamentally alter how global health relates to these entities.

## Footnotes

† Co-creation is the creation of solutions, through a design process, with users or stakeholders. Guided co-creation is led by a design facilitator who has significant experience in the geography and/or area of global health.

## Notes

**Peer Reviewed**
